# Does Socioeconomic Status Moderate the Association Between Metabolic Syndrome and Cognitive Decline? A Moderated Mediation Analysis Using the China Health and Retirement Longitudinal Study (CHARLS)

**DOI:** 10.21203/rs.3.rs-10497702/v1

**Published:** 2026-09-11

**Authors:** Yifan Liang, Shihuan Zhong

**Affiliations:** Traditional Chinese Medicine Hospital of Yulin; Guangxi Medical University

**Keywords:** metabolic syndrome, cognitive decline, socioeconomic status, moderated mediation, C-reactive protein, CHARLS

## Abstract

**Background:**

Metabolic syndrome (MetS) has been associated with cognitive decline, yet the role of socioeconomic status (SES) in modifying this association through inflammatory pathways remains unclear. This study investigated whether SES moderates the mediating effect of C-reactive protein (CRP) in the MetS–cognitive decline pathway.

**Methods:**

We used longitudinal data from the China Health and Retirement Longitudinal Study (CHARLS) spanning 2011–2018. The final analytic sample comprised 2,357 participants (aged ≥ 45 years, baseline stroke-free, with complete MetS, cognitive function, SES, and 2015 CRP data). MetS was defined using the IDF Asian criteria. Cognitive decline was operationalized as the difference between 2011 and 2018 total cognitive scores. An SES index was constructed from standardized education years and household income. A moderated mediation model with log(CRP + 1) at 2015 as the mediator was tested using 5,000 bootstrap resamples. Sensitivity analyses included continuous MetS component count, education-only SES, and exclusion of CRP > 10 mg/L.

**Results:**

Among 2,357 participants (29.6% with MetS, mean age 58.2 years), MetS significantly predicted higher CRP levels (β = 0.120, *p* < 0.001), but CRP was not significantly associated with cognitive decline (β = 0.041, *p* = 0.798). The Index of Moderated Mediation was non-significant (IMM = − 0.0003, 95% CI [− 0.014, 0.013]). Stratified analyses revealed no significant indirect effects through CRP in any SES stratum. All sensitivity analyses yielded consistent null findings. Notably, SES emerged as the strongest independent predictor of cognitive decline (β = −1.89, *p* < 0.001).

**Conclusions:**

Although MetS elevates systemic inflammation as indexed by CRP, the CRP pathway does not significantly mediate the effect of MetS on cognitive decline, and this indirect effect is not moderated by SES. SES independently and strongly predicts cognitive decline trajectories, suggesting that socioeconomic factors may influence cognitive aging through pathways beyond metabolic inflammation.

## 1. Introduction

Metabolic syndrome (MetS)—a clustering of central obesity, hypertension, hyperglycemia, hypertriglyceridemia, and low high-density lipoprotein cholesterol—is increasingly prevalent worldwide [1]. In China, an estimated 24–33% of adults meet MetS criteria, and rates continue to climb with population aging [2]. Epidemiological studies have linked MetS to faster cognitive decline and higher dementia risk [3–5] a meta-analysis estimated that MetS raises the odds of cognitive impairment by roughly 20–50% [6].

How MetS damages cognition is still not fully understood. The inflammation hypothesis holds that MetS-driven low-grade systemic inflammation accelerates cognitive decline through blood–brain barrier disruption, neuroinflammation, and neuronal injury [7, 8]. C-reactive protein (CRP), a widely measured marker of systemic inflammation, has been examined as a potential mediator of the MetS–cognition relationship [9], though results have been mixed—some prospective studies report significant associations [10] while others do not [11].

Socioeconomic status (SES) is a powerful social determinant of health, linked to morbidity, mortality, and cognitive aging [12]. Low SES consistently predicts higher MetS prevalence, greater inflammatory burden, and steeper cognitive decline [13–15]. But SES may play a role beyond simple confounding. Three theoretical frameworks suggest SES could moderate the MetS–inflammation–cognition pathway. The buffering hypothesis proposes that higher SES provides protective resources—better healthcare, health literacy, coping capacity—that blunt the cognitive toll of metabolic dysfunction [16]. The cumulative disadvantage hypothesis, by contrast, argues that low SES and MetS jointly worsen cognitive decline by restricting health behaviors and access to timely care [17]. The differential susceptibility hypothesis further suggests that the biological imprint of MetS-related risk differs across SES strata, producing heterogeneous inflammation–cognition associations [18].

No study has used moderated mediation analysis to test whether SES changes the indirect effect of MetS on cognitive decline through inflammatory pathways. This is worth examining for two reasons. First, identifying SES-dependent mediation could guide more targeted interventions. Second, quantifying the independent and interactive contributions of metabolic and socioeconomic factors can clarify how much of cognitive aging is driven by biology versus social circumstance.

We therefore set out to answer three questions: (1) Does MetS affect cognitive decline indirectly through elevated CRP? (2) Does this indirect effect differ by SES level? (3) What is the independent effect of SES on cognitive decline trajectories? We used four waves of nationally representative CHARLS data in a time-separated moderated mediation design to strengthen causal inference.

## 2. Methods

### 2.1 Data Source and Study Design

Data were drawn from the China Health and Retirement Longitudinal Study (CHARLS) [19], a nationally representative longitudinal survey of Chinese adults aged 45 years and older. The baseline survey was conducted in 2011, with follow-up waves every 2–3 years. CHARLS employs multistage probability-proportional-to-size sampling across 450 villages/urban communities in 28 provinces, achieving high response rates (> 80%). The study received ethical approval from the Peking University Institutional Review Board, and all participants provided written informed consent.

We adopted a longitudinal moderated mediation design with temporal separation of exposure, mediator, and outcome: (1) exposure and moderator (MetS, SES) measured at T1 (2011); (2) mediator (CRP) measured at T2 (2015); and (3) outcome (cognitive decline) derived from cognitive assessments at T1 (2011) and T3 (2018). This design mitigates reverse causality concerns inherent in cross-sectional mediation models. Data were obtained from three CHARLS datasets: the Harmonized CHARLS D dataset (Waves 2011, 2013, 2015, 2018; N = 25,586), the 2011 Blood biomarker dataset (N = 13,420), and the 2015 Blood biomarker dataset (Blood_20140429; N = 11,847).

### 2.2 Study Population

The sample selection flow was as follows. From the 25,586 individuals in the Harmonized CHARLS D dataset, we sequentially restricted to: age ≥ 45 years (n = 16,931), complete MetS component data (n = 13,022), complete baseline cognitive assessment (n = 10,042), complete 2018 cognitive follow-up (n = 5,455), complete SES data (n = 3,224), no baseline stroke (n = 3,151), and available 2015 CRP data (n = 2,357). The final analytic sample thus comprised 2,357 participants.

### 2.3 Variables

#### 2.3.1 Exposure: Metabolic Syndrome

MetS was defined according to the 2006 International Diabetes Federation (IDF) Asian criteria [20], requiring central obesity (waist circumference ≥ 90 cm for men or ≥ 80 cm for women) plus any two of the following: (1) elevated triglycerides (≥ 150 mg/dL or lipid-lowering treatment); (2) reduced HDL-C (< 40 mg/dL for men or < 50 mg/dL for women); (3) elevated blood pressure (systolic ≥ 130 mmHg or diastolic ≥ 85 mmHg or self-reported hypertension); and (4) elevated fasting glucose (≥ 100 mg/dL or self-reported diabetes). Waist circumference, blood pressure, and fasting blood biomarkers were measured by trained staff using standardized protocols. We also calculated a continuous MetS component score (0–5) for sensitivity analyses.

#### 2.3.2 Mediator: C-Reactive Protein

High-sensitivity CRP (mg/L) was assayed from fasting venous blood samples collected during the 2015 wave. CRP values were natural-log-transformed [log(CRP + 1)] to approximate normality. This time point was selected to ensure temporal precedence between the exposure (2011) and the mediator (2015) while allowing sufficient time for the mediator to influence the outcome (cognitive decline through 2018).

#### 2.3.3 Moderator: Socioeconomic Status Index

An SES composite index was constructed from two standardized indicators measured at baseline (2011): (1) education years, converted from CHARLS categorical education levels to continuous years (illiterate = 0, incomplete primary = 3, Sishu = 3, primary = 6, middle school = 9, high school/vocational = 12, college = 15, bachelor’s = 16, postgraduate = 19); and (2) household income, log-transformed. The SES index was computed as SES = [z (education years) + z(log(household income + 1))] / 2. Participants were further classified into Low, Medium, and High SES groups based on tertiles of the index for stratified analyses.

#### 2.3.4 Outcome: Cognitive Decline

CHARLS cognitive assessment comprises four subscales: (1) Word Recall (0–20), summing immediate and delayed recall of 10 words; (2) Serial 7 Subtraction (0–5), counting correct responses in five consecutive subtractions; (3) Orientation (0–4), assessing awareness of year, month, day, and season; and (4) Figure Drawing (0–1), requiring replication of two overlapping pentagons. The total cognitive score ranges from 0 to 30. Cognitive decline was defined as the difference between 2011 and 2018 total cognitive scores, with positive values indicating decline.

#### 2.3.5 Covariates

All models were adjusted for the following baseline (2011) covariates: age (years), sex (female = 1), body mass index (BMI, kg/m^2^), depressive symptoms (CESD-10, range 0–30), smoking history (ever smoker = 1), alcohol consumption history (ever drinker = 1), and baseline total cognitive score. The inclusion of baseline cognitive score accounts for regression to the mean effects[21].

### 2.4 Statistical Analysis

Descriptive statistics were presented as mean ± standard deviation (SD) for continuous variables and frequency (percentage) for categorical variables, stratified by MetS status. Group differences were tested using independent-samples t-tests for continuous variables and chi-square tests for categorical variables.

The core moderated mediation model comprised two linear regression equations [20]. Model 1 (Path a) regressed CRP (M) on MetS (X), SES (W), the X × W interaction, and covariates. Model 2 (Paths b and c′) regressed cognitive decline (Y) on MetS (X), CRP (M), SES (W), M × W, X × W, and covariates. The conditional indirect effect of MetS on cognitive decline through CRP at a given SES level was computed as (a_1_ + a_3_W) × (b_1_ + b_3_W). The Index of Moderated Mediation (IMM) quantified whether the indirect effect varied linearly as a function of SES [22].

Statistical inference for indirect effects was based on 5,000 nonparametric bootstrap resamples with bias-corrected percentile 95% confidence intervals. An indirect effect was considered statistically significant if the 95% CI excluded zero.

Three sensitivity analyses were conducted: (SA1) replacing the binary MetS variable with the continuous MetS component count (0–5); (SA2) using education-only tertiles as the SES measure to isolate the cognitive dimension of SES; and (SA3) excluding participants with CRP > 10 mg/L (n = 2,293 retained) to minimize confounding by acute infection or inflammation. All analyses were performed using Python 3.12 with pandas, numpy, scipy and statsmodels. Statistical significance was set at α = 0.05 (two-tailed).

## 3. Results

### 3.1 Sample Characteristics

The final analytic sample comprised 2,357 participants, of whom 696 (29.6%) met the MetS criteria. The mean age was 58.2 years (SD = 7.8). Table 1 presents baseline characteristics stratified by MetS status. Compared with the non-MetS group, MetS participants were slightly older (58.8 vs. 57.9 years, *p* = 0.006), had significantly higher BMI, waist circumference, blood pressure, fasting glucose, and triglycerides, and lower HDL-C (all *p* < 0.001). The MetS group had a higher proportion of women (61.4% vs. 37.7%, *p* < 0.001) and lower proportions of ever smokers (33.3% vs. 49.0%, *p* < 0.001) and ever drinkers (33.8% vs. 46.7%, *p* < 0.001). Baseline hs-CRP was significantly higher in the MetS group (2.92 vs. 2.03 mg/L, *p* < 0.001). Notably, baseline and 2018 cognitive scores, as well as the magnitude of cognitive decline, did not differ significantly between groups (all *p* > 0.05).

### 3.2 Moderated Mediation Analysis

In Model 1 (Path a), MetS significantly predicted higher 2015 log(CRP + 1) levels (β = 0.120, SE = 0.028, *p* < 0.001, 95% CI [0.065, 0.175]), confirming that MetS is associated with elevated systemic inflammation. The SES × MetS interaction term was not significant (β = −0.003, *p* = 0.944), indicating that the MetS–CRP association was consistent across SES levels. Model 1 explained 5.4% of the variance in CRP (R^2^ = 0.054).

In Model 2 (Paths b and c′), log(CRP + 1) was not significantly associated with cognitive decline (β = 0.041, SE = 0.158, *p* = 0.798, 95% CI [− 0.269, 0.350]). The direct effect of MetS on cognitive decline was also non-significant (β = −0.264, *p* = 0.220). SES emerged as the strongest predictor: each standard deviation increase in the SES index was associated with 1.89 points less cognitive decline (β = −1.891, SE = 0.266, *p* < 0.001, 95% CI [− 2.412, − 1.370]). The CRP × SES (p = 0.754) and MetS × SES (p = 0.578) interaction terms were both non-significant. Model 2 explained 23.3% of the variance in cognitive decline (R^2^ = 0.233). Baseline cognitive score was positively associated with greater decline (β = 0.544, *p* < 0.001), consistent with regression to the mean effects. Full model coefficients are presented in Table 2.

### 3.3 Bootstrap Conditional Indirect Effects

Bootstrap-derived conditional indirect effects of MetS on cognitive decline through CRP were computed at three SES levels. At Low SES (− 1SD), the indirect effect was 0.0050 (95% CI [− 0.036, 0.049]); at Mean SES, 0.0057 (95% CI [− 0.035, 0.046]); and at High SES (+ 1SD), 0.0054 (95% CI [− 0.034, 0.049]). None of these indirect effects were statistically significant. The Index of Moderated Mediation was − 0.0003 (95% CI [− 0.014, 0.013]), confirming that the indirect effect of MetS on cognitive decline through CRP did not vary as a function of SES (Table 3).

### 3.4 SES-Stratified Mediation Analysis

Stratified mediation analyses by SES tertiles are presented in Table 4. Path a (MetS → CRP) was significant only in the High SES group (β = 0.136, *p* < 0.001, n = 1,140), whereas it did not reach significance in the Low SES (β = 0.137, *p* > 0.05, n = 382) or Medium SES (β = 0.088, *p* > 0.05, n = 835) groups—likely reflecting reduced statistical power in the smaller Low SES stratum. Path b (CRP → cognitive decline) was non-significant across all three SES strata. Correspondingly, no indirect effect was statistically significant in any stratum: Low SES, 0.023 (95% CI [− 0.107, 0.188]); Medium SES, 0.008 (95% CI [− 0.043, 0.068]); High SES, 0.003 (95% CI [− 0.064, 0.067]).

### 3.5 Sensitivity Analyses

All three sensitivity analyses corroborated the main findings. Neither the continuous MetS component score (SA1), education-only SES (SA2), nor exclusion of participants with CRP > 10 mg/L (SA3) yielded significant indirect effects at any SES level. The null finding regarding CRP-mediated indirect effects and the absence of SES moderation were robust across all alternative specifications.

## 4. Discussion

### 4.1 Summary of Principal Findings

We used nationally representative longitudinal CHARLS data to test whether SES moderates the indirect effect of MetS on cognitive decline through systemic inflammation (CRP). Three main findings emerged. First, MetS predicted higher CRP—confirming the low-grade chronic inflammation hypothesis—but CRP did not mediate the MetS–cognitive decline link; indirect effects were null at all SES levels. Second, the Index of Moderated Mediation was non-significant, providing no evidence that SES modifies the CRP-mediated pathway. Third, SES was by far the strongest predictor of cognitive decline, with a standardized effect nearly an order of magnitude larger than those of MetS or CRP. These results held across multiple sensitivity analyses, including alternative MetS and SES definitions and exclusion of participants with elevated CRP.

### 4.2 MetS–Inflammation Pathway

The significant MetS–CRP association (β = 0.120, p < 0.001) is consistent with the large existing literature on metabolic dysregulation and systemic inflammation. The effect size is comparable to meta-analytic estimates from Western cohorts [22]. One result worth noting: in stratified analyses, the MetS–CRP link was significant only in the High SES group (β = 0.136, p < 0.001, n = 1,140) and not in the Low (n = 382) or Medium (n = 835) SES strata. There are several possible explanations for this reverse gradient—metabolically driven inflammation most detectable among the advantaged. The Low SES stratum had limited statistical power, which likely inflated standard errors. Competing inflammatory stimuli (poor living conditions, environmental exposures, chronic psychosocial stress) may dominate the inflammatory profile in disadvantaged populations, partly masking MetS-specific effects on CRP. Selective survival may have differentially removed high-risk individuals from lower SES strata.

### 4.3 Inflammation–Cognition Pathway: A Null Finding

The null CRP–cognitive decline association (β = 0.041, p = 0.798) differs from some reports [10] but aligns with others [11]. Several factors may explain this. The temporal design—CRP measured in 2015, cognitive decline assessed over a 2011–2018 window—may be suboptimal; a single mid-point CRP measurement probably captures chronic inflammatory burden poorly, especially for neurodegenerative processes that unfold over decades. CRP is a non-specific acute-phase reactant and may not reflect the neuroinflammatory pathways most relevant to cognitive decline—those involving interleukin-6, tumor necrosis factor-α, or soluble adhesion molecules [23]. The inflammation–cognition relationship could also be non-linear (U-shaped or threshold effects), which linear mediation models would miss. Finally, the CHARLS cognitive battery, though covering multiple domains, may lack sensitivity to detect subtle, inflammation-related changes over seven years [24].

### 4.4 The most consequential finding is the size of the SES effect on cognitive decline (β = −1.89, p < 0.001).

One standard deviation higher SES was associated with roughly 1.9 points less decline—an effect far larger than those of MetS or CRP. This effect persisted after adjustment for all measured confounders and was independent of the MetS–CRP pathway. The result is consistent with the Social Stratification Theory and the cognitive reserve framework [21]: socioeconomic advantage works through multiple reinforcing channels—education building cognitive reserve, occupational complexity sustaining mental engagement, and economic resources enabling healthier behaviors and better healthcare. The implication is straightforward: the ‘metabolic-cognitive’ risk attributed to MetS may in large part be carried by socioeconomic rather than purely biological pathways. Targeting metabolic risk factors without addressing socioeconomic inequalities may therefore yield only partial cognitive health benefits at the population level.

The non-significant Index of Moderated Mediation (IMM = − 0.0003) indicates that the CRP-mediated indirect effect of MetS on cognitive decline did not vary across SES levels. This null finding may reflect a genuine absence of SES moderation in the CRP pathway, or it may result from the combination of a weak Path b (CRP → cognition) and limited sample size in the lower SES strata, which constrained the power to detect interaction effects. The buffering, cumulative disadvantage, and differential susceptibility hypotheses remain theoretically plausible for alternative pathways—such as cerebrovascular damage [25], neurotrophic factors, or structural brain changes—that could not be tested with the available CHARLS biomarkers. Future studies employing multi-marker panels and multimodal neuroimaging are needed to extend this investigation to pathways beyond systemic inflammation.

### 4.5 Strengths and Limitations

This study has several strengths. It is the first to apply moderated mediation to the question of whether SES modifies the MetS–inflammation–cognitive decline pathway. The time-separated longitudinal design—with exposure, mediator, and outcome measured at distinct time points spanning seven years—strengthens causal inference relative to cross-sectional work [26]. CHARLS provides a nationally representative Chinese cohort, enhancing generalizability to aging populations in low- and middle-income settings where MetS is rising fastest. The multidimensional SES index (education plus income) captures social stratification more comprehensively than single-indicator measures. The set of sensitivity analyses reinforces confidence in the core conclusions.

Several limitations must be acknowledged. First, CRP was available at only a single time point (2015) after multiple waves of sample attrition; the reduction from 25,586 to 2,357 participants (90.8% attrition) introduces potential selection bias, as retained individuals are likely healthier (healthy survivor effect), which may attenuate effect estimates toward the null. Second, CRP alone cannot capture the complexity of MetS-related neuroinflammatory pathways; the true mediating role of inflammation may be underestimated. Third, the cognitive assessment battery, while validated for CHARLS, may lack sensitivity to subtle subclinical cognitive changes. Fourth, unmeasured confounders—including APOE ε4 genotype, dietary quality, physical activity, social engagement, and air pollution exposure—could bias the estimated associations. Fifth, SES was treated as time-invariant (measured at 2011), whereas retirement, income changes, and other life transitions during the 7-year follow-up may have altered individuals’ socioeconomic circumstances. Sixth, the focus on cognitive decline as a continuous measure, while maximizing statistical power, does not address the clinical relevance of incident cognitive impairment or dementia.

## 5. Conclusions

In this nationally representative longitudinal cohort of middle-aged and older Chinese adults, MetS raised systemic inflammation as measured by CRP, but CRP did not mediate the effect of MetS on cognitive decline. SES did not moderate this indirect effect. However, SES alone was the dominant predictor of cognitive decline trajectories, with an effect far exceeding those of metabolic and inflammatory risk factors. These findings suggest that while metabolic health and inflammation matter for cognitive aging, socioeconomic factors may be the stronger determinants of how cognition changes in later life. From a public health standpoint, reducing social inequalities—improving education and economic security—could yield cognitive health gains that match or exceed those from metabolic risk factor management. Future work should examine SES moderation across multiple biomarker pathways, use longer follow-up with repeated biomarker measures, and incorporate clinical cognitive outcomes to better characterize the interplay between social stratification and metabolic-cognitive health.

## Figures and Tables

**Figure 1:**
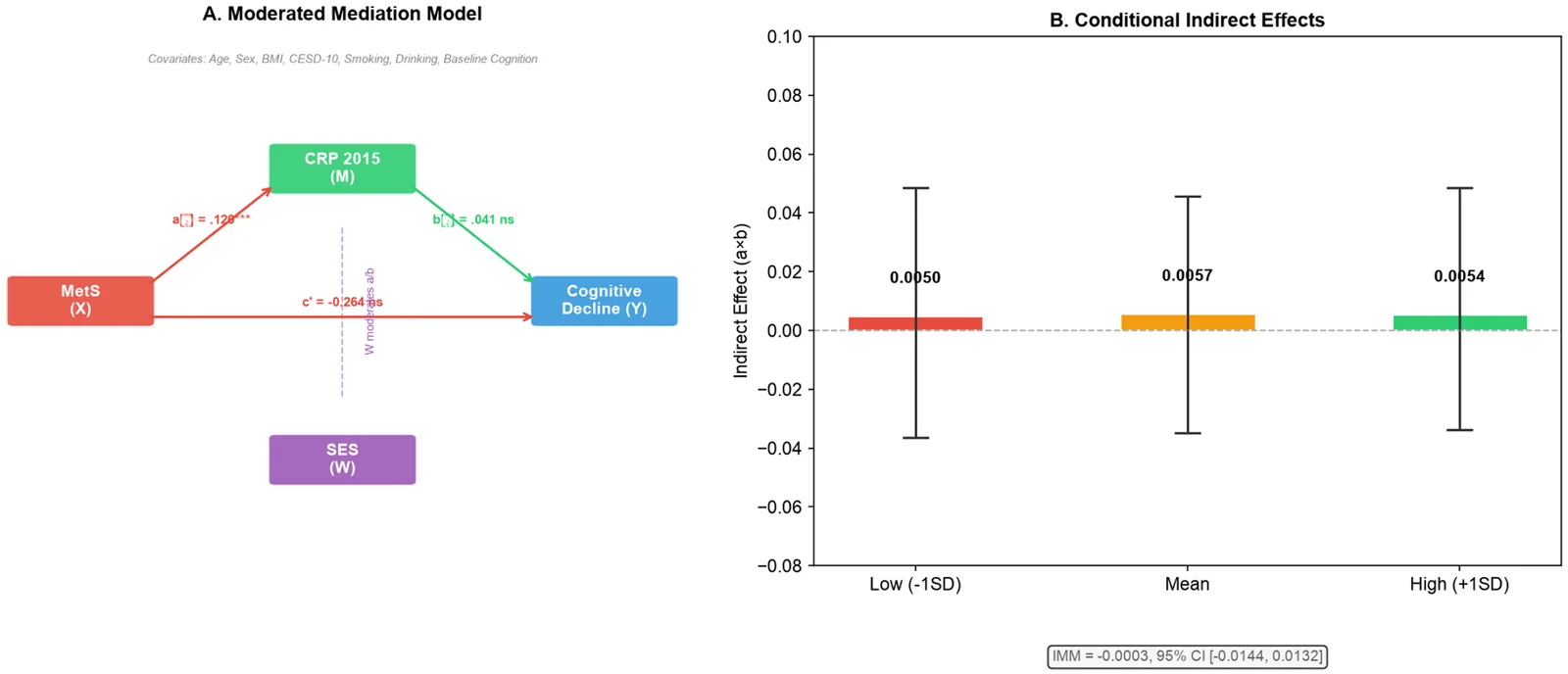
Legend not included with this version

**Figure 2:**
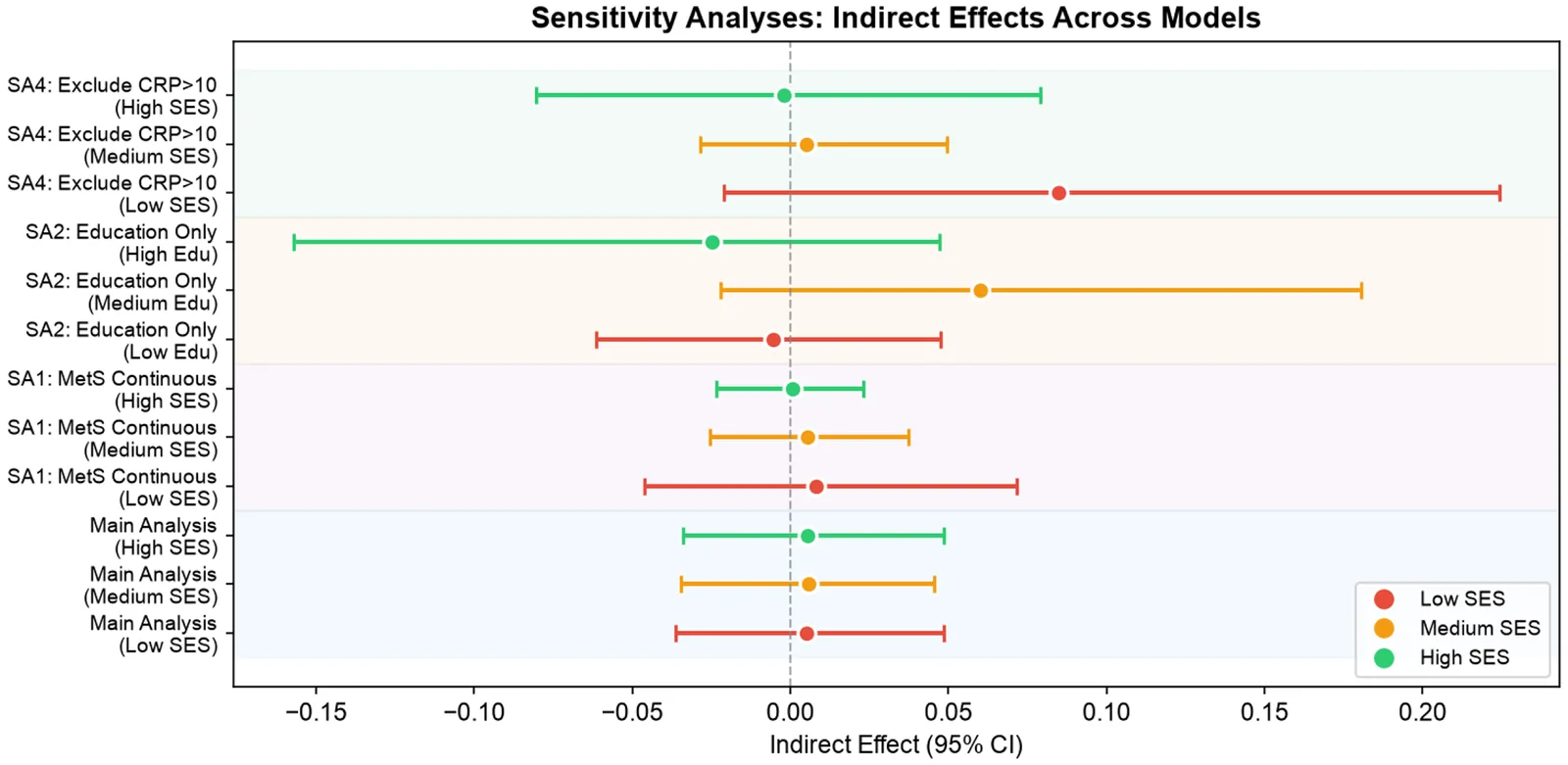
Legend not included with this version

**Figure 3:**
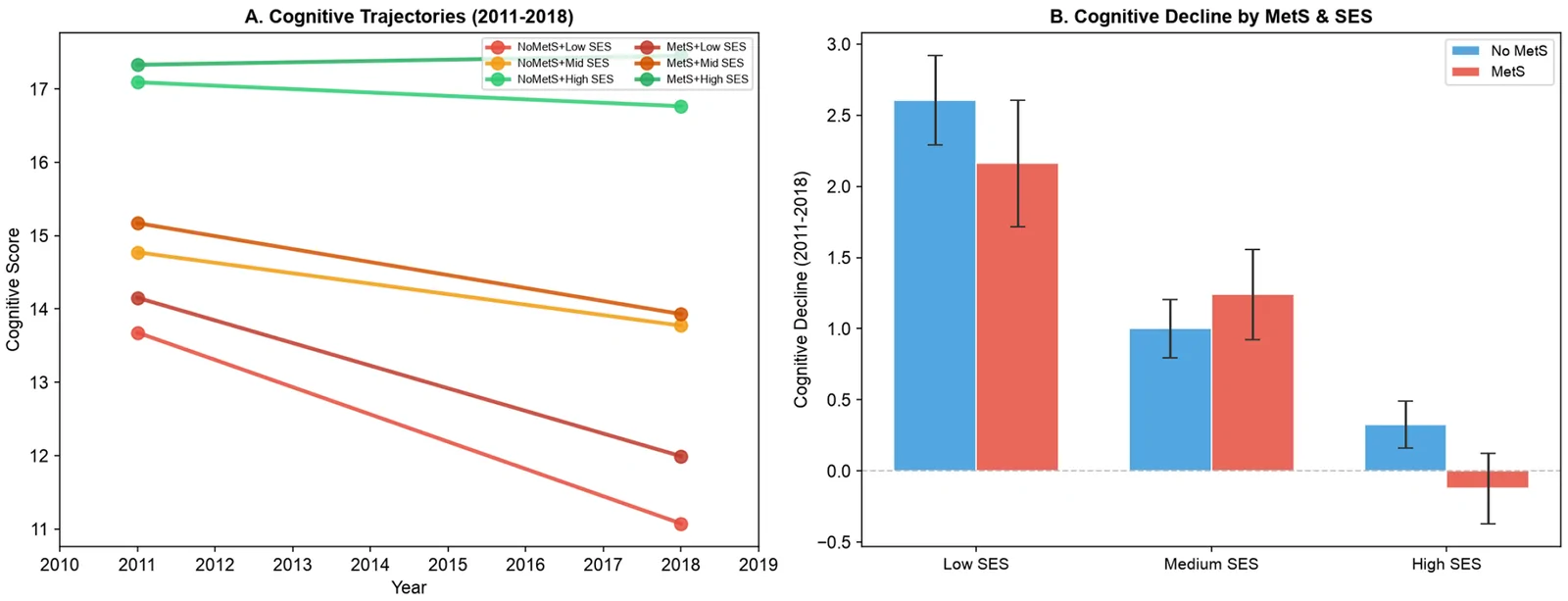
Legend not included with this version

**Figure 4:**
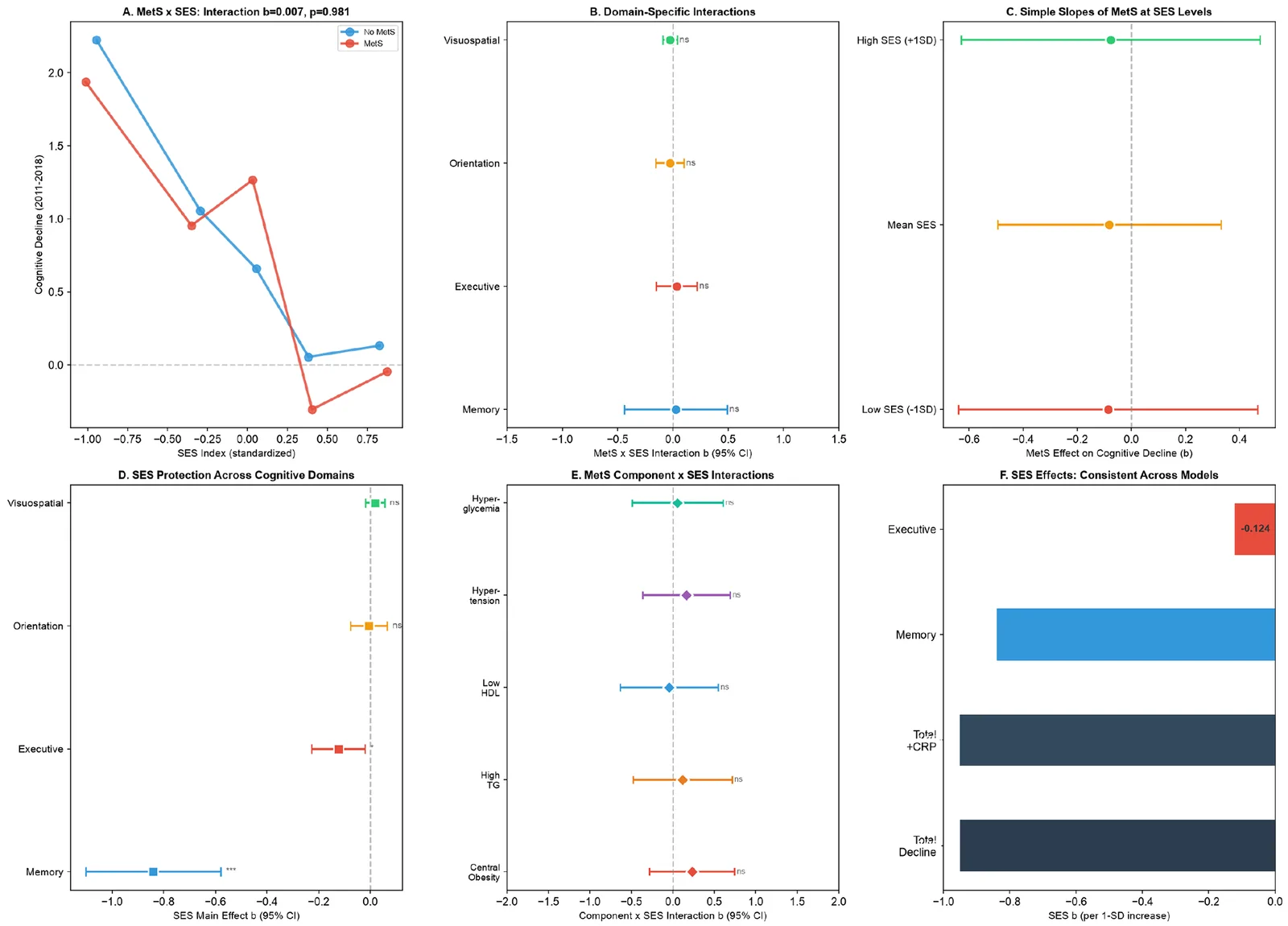
Legend not included with this version

**Figure 5:**
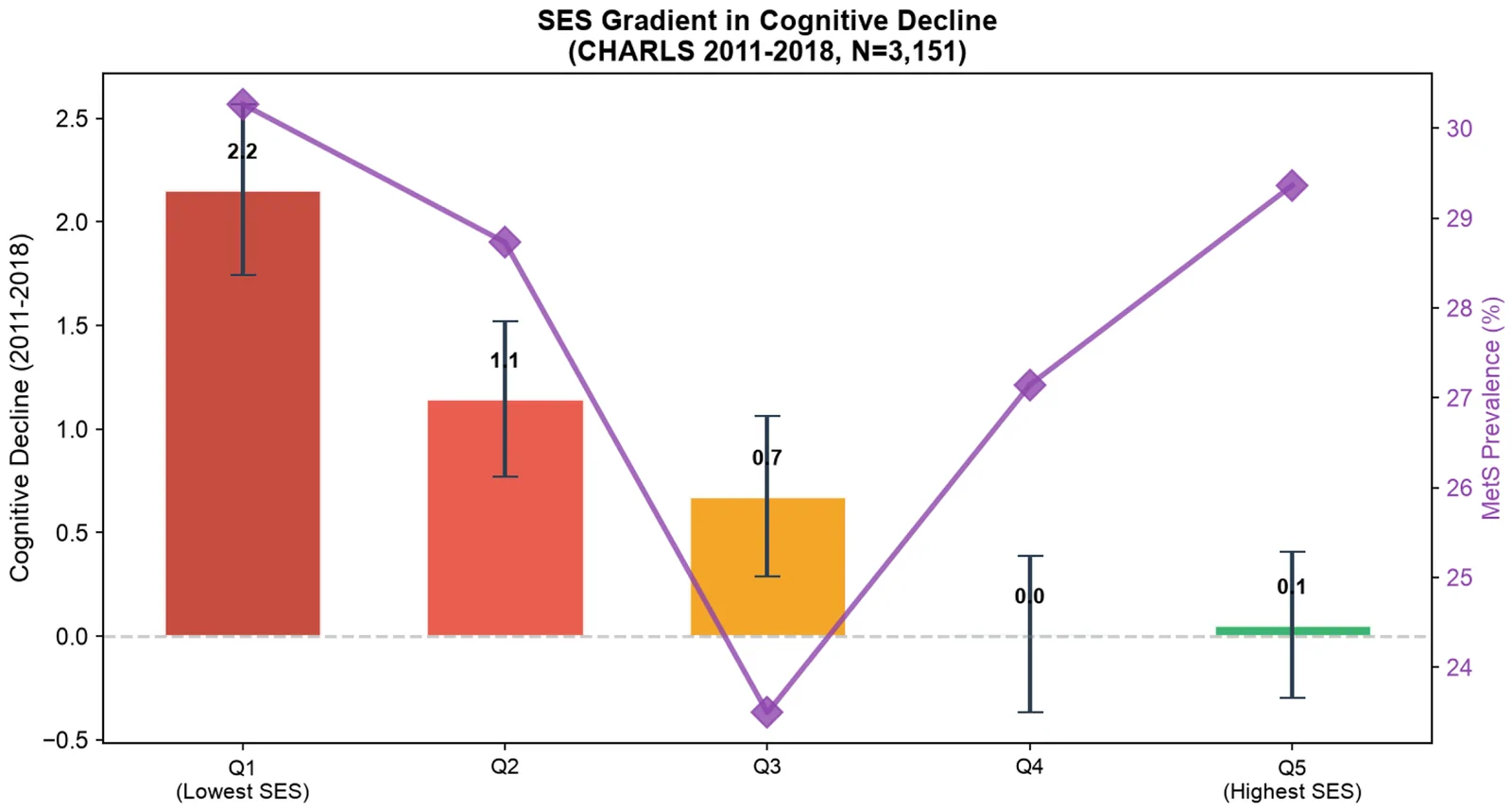
Legend not included with this version

**Table 1:** Baseline Characteristics Stratified by Metabolic Syndrome Status

Characteristic	Total (N = 2,357)	No MetS (n = 1,661)	MetS (n = 696)	Test Statistic	p
Age (years)	58.15 (7.79)	57.87 (7.88)	58.84 (7.55)	t = 2.76	0.006
BMI (kg/m^2^)	23.74 (3.62)	22.89 (3.32)	25.76 (3.44)	t = 18.85	< 0.001
Waist circumference (cm)	85.95 (9.99)	83.35 (9.29)	92.15 (8.74)	t = 21.25	< 0.001
SBP (mmHg)	131.64 (21.63)	128.38 (20.82)	139.41 (21.83)	t = 11.45	< 0.001
DBP (mmHg)	76.83 (12.66)	75.18 (12.30)	80.76 (12.64)	t = 9.96	< 0.001
Fasting glucose (mg/dL)	99.87 (24.08)	94.86 (18.56)	111.82 (31.31)	t = 13.22	< 0.001
Triglycerides (mg/dL)	136.02 (86.42)	107.77 (56.36)	203.42 (107.02)	t = 21.51	< 0.001
HDL-C (mg/dL)	51.13 (10.60)	53.63 (10.46)	45.18 (8.28)	t = − 20.80	< 0.001
hs-CRP (mg/L)	2.29 (3.03)	2.03 (3.00)	2.92 (3.00)	t = 6.52	< 0.001
Baseline cognition	15.82 (4.20)	15.74 (4.23)	16.00 (4.13)	t = 1.40	0.161
2018 cognition	14.95 (5.26)	14.82 (5.27)	15.24 (5.24)	t = 1.78	0.076
Cognitive decline	0.87 (4.89)	0.92 (4.92)	0.76 (4.81)	t = − 0.71	0.481
CESD-10	7.94 (6.04)	7.94 (6.02)	7.92 (6.09)	t = − 0.07	0.943
Education (years)	6.43 (3.63)	6.47 (3.61)	6.32 (3.66)	t = − 0.92	0.358
Female, n (%)	1,053 (44.7%)	626 (37.7%)	427 (61.4%)	χ^2^ = 111.8	< 0.001
Ever smoker, n (%)	1,046 (44.4%)	814 (49.0%)	232 (33.3%)	χ^2^ = 48.7	< 0.001
Ever drinker, n (%)	1,010 (42.9%)	775 (46.7%)	235 (33.8%)	χ^2^ = 33.0	< 0.001

Note: Values are mean (SD) unless otherwise indicated. BMI = body mass index; SBP = systolic blood pressure; DBP = diastolic blood pressure; HDL-C = high-density lipoprotein cholesterol; hs-CRP = high-sensitivity C-reactive protein; CESD-10 = 10-item Center for Epidemiologic Studies Depression scale.

**Table 2:** Moderated Mediation Model: Regression Coefficients Panel A: Model 1 — MetS → log(CRP + 1) 2015 (Path a)

Variable	b (SE)	t	*p*	95% CI Lower	95% CI Upper
MetS (X)	0.120 (0.028)	4.285	< 0.001	0.065	0.175
SES (W)	0.002 (0.023)	0.081	0.936	−0.043	0.046
MetS × SES	−0.003 (0.039)	−0.071	0.944	−0.079	0.074
**Panel B: Model 2 — CRP + MetS → Cognitive Decline (Paths b and c’)**					
Variable	b (SE)	t	*p*	95% CI Lower	95% CI Upper
MetS (X)	−0.264 (0.215)	−1.228	0.220	−0.684	0.157
log CRP (M)	0.041 (0.158)	0.257	0.798	−0.269	0.350
**SES (W)**	**−1.891 (0.266)**	**−7.116**	**< 0.001**	**−2.412**	**−1.370**
CRP × SES	−0.078 (0.248)	−0.314	0.754	−0.564	0.409
MetS × SES	−0.167 (0.300)	−0.556	0.578	−0.756	0.422

R^2^ = 0.054. Adjusted for age, sex, BMI, CESD-10, smoking, alcohol, baseline cognition.

R^2^ = 0.233. Adjusted for age, sex, BMI, CESD-10, smoking, alcohol, baseline cognition.

**Table 3:** Bootstrap Conditional Indirect Effects and Index of Moderated Mediation

SES Level	Indirect Effect	Boot SE	95% CI Lower	95% CI Upper	Significant
Low (− 1SD)	0.0050	0.0210	−0.036	0.049	No
Mean	0.0057	0.0199	−0.035	0.046	No
High (+ 1SD)	0.0054	0.0202	−0.034	0.049	No

Index of Moderated Mediation (IMM) = − 0.0003, Boot SE = 0.0065, 95% CI [− 0.014, 0.013]. Based on 5,000 bootstrap resamples.

**Table 4:** SES-Stratified Mediation Analysis

SES Group	n	Path a (MetS→CRP)	Path b (CRP→Decline)	Indirect Effect	Bootstrap 95% CI
Low SES	382	0.137 (p > 0.05)	0.131 (p > 0.05)	0.023	[− 0.107, 0.188]
Medium SES	835	0.088 (p > 0.05)	0.091 (p > 0.05)	0.008	[− 0.043, 0.068]
High SES	1,140	0.136 (p < 0.001)	0.028 (p > 0.05)	0.003	[− 0.064, 0.067]

Note: All models adjusted for age, sex, BMI, CESD-10, smoking, alcohol, and baseline cognition.

## Data Availability

The CHARLS datasets analyzed during the current study are publicly available at the CHARLS website (http://charls.pku.edu.cn/). Access requires registration and submission of a data use agreement. The analytic code is available from the corresponding author upon reasonable request.
